# Supplementary material for: Leaf shedding as an anti-bacterial defense in Arabidopsis cauline leaves
Source: PLoS Genet. 2017 Dec 18;13(12):e1007132. doi: 10.1371/journal.pgen.1007132 (PMC5749873; doi:10.1371/journal.pgen.1007132)
Supplement: S1 Fig — The top panel shows the second cauline leaf on the primary inflorescence. The bottom panel is a magnification of the circled area in the top panel. The cauline leaf abscission zone enables cauline leaves to be shed. (PDF) [file pgen.1007132.s001.pdf]

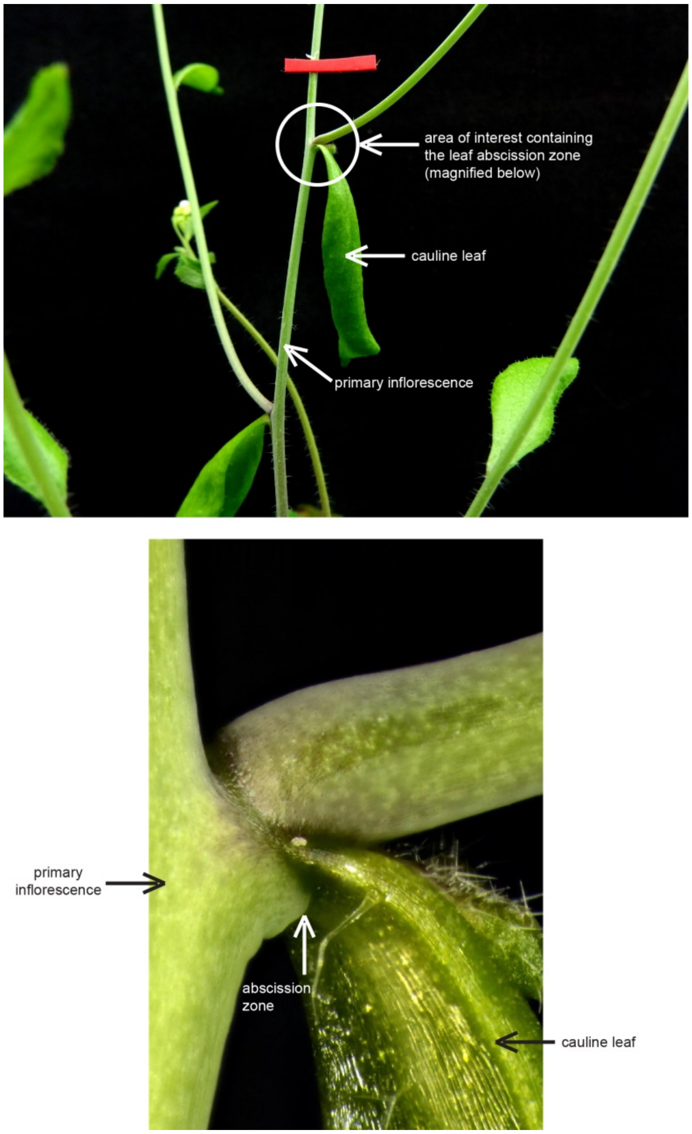

S1 Fig. **The cauline leaf abscission system**. The top panel shows the second cauline leaf on the primary inflorescence. The bottom panel is a magnification of the circled area in the top panel. The cauline leaf abscission zone enables cauline leaves to be shed.
