# Supplementary material for: Leaf shedding as an anti-bacterial defense in Arabidopsis cauline leaves
Source: PLoS Genet. 2017 Dec 18;13(12):e1007132. doi: 10.1371/journal.pgen.1007132 (PMC5749873; doi:10.1371/journal.pgen.1007132)
Supplement: S2 Fig — Bacterial enumeration in (A) cauline leaves and (B) rosette leaves from flowering plants grown in 16 h light / 8 h dark with 50–65% relative humidity. (C) Bacterial enumeration of non-flowering plants grown in 8 h light / 16 h dark with ≥ 75% relative humidity. (PDF) [file pgen.1007132.s002.pdf]

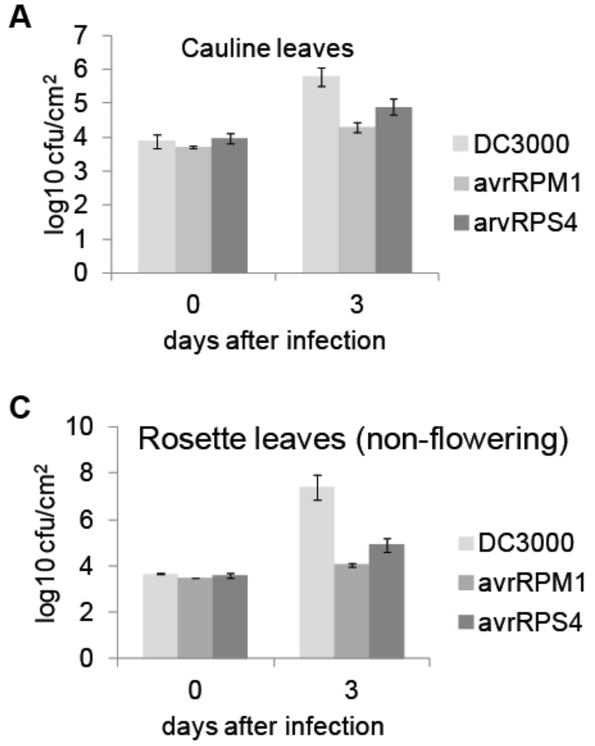

S2 Fig. Flowering plants are more resistant to *Pst* than non-flowering plants. Bacterial enumeration in (A) cauline leaves and (B) rosette leaves from flowering plants grown in 16 h light / 8 h dark with 50-65% relative humidity. (C) Bacterial enumeration of non-flowering plants grown in 8 h light / 16 h dark with ≥ 75% relative humidity.
