# Supplementary material for: Leaf shedding as an anti-bacterial defense in Arabidopsis cauline leaves
Source: PLoS Genet. 2017 Dec 18;13(12):e1007132. doi: 10.1371/journal.pgen.1007132 (PMC5749873; doi:10.1371/journal.pgen.1007132)
Supplement: S3 Fig — Note the left side of the cauline leaf has peeled off of the abscission zone while the right side of the cauline leaf remains attached. (PDF) [file pgen.1007132.s003.pdf]

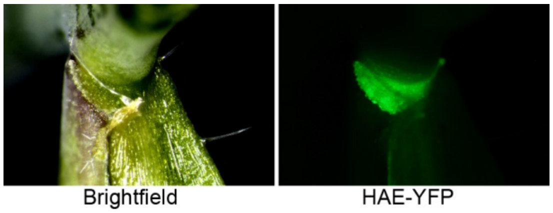

S3 Fig. View from the bottom of a cauline leaf infected on the left quarter which is touching the AZ. Note the left side of the cauline leaf has peeled off of the abscission zone while the right side of the cauline leaf remains attached.
