# Supplementary material for: Leaf shedding as an anti-bacterial defense in Arabidopsis cauline leaves
Source: PLoS Genet. 2017 Dec 18;13(12):e1007132. doi: 10.1371/journal.pgen.1007132 (PMC5749873; doi:10.1371/journal.pgen.1007132)
Supplement: S4 Fig — Blue arrow indicates the area where cell separation has occurred. Red arrow indicates swollen abscission zone cells. (PDF) [file pgen.1007132.s004.pdf]

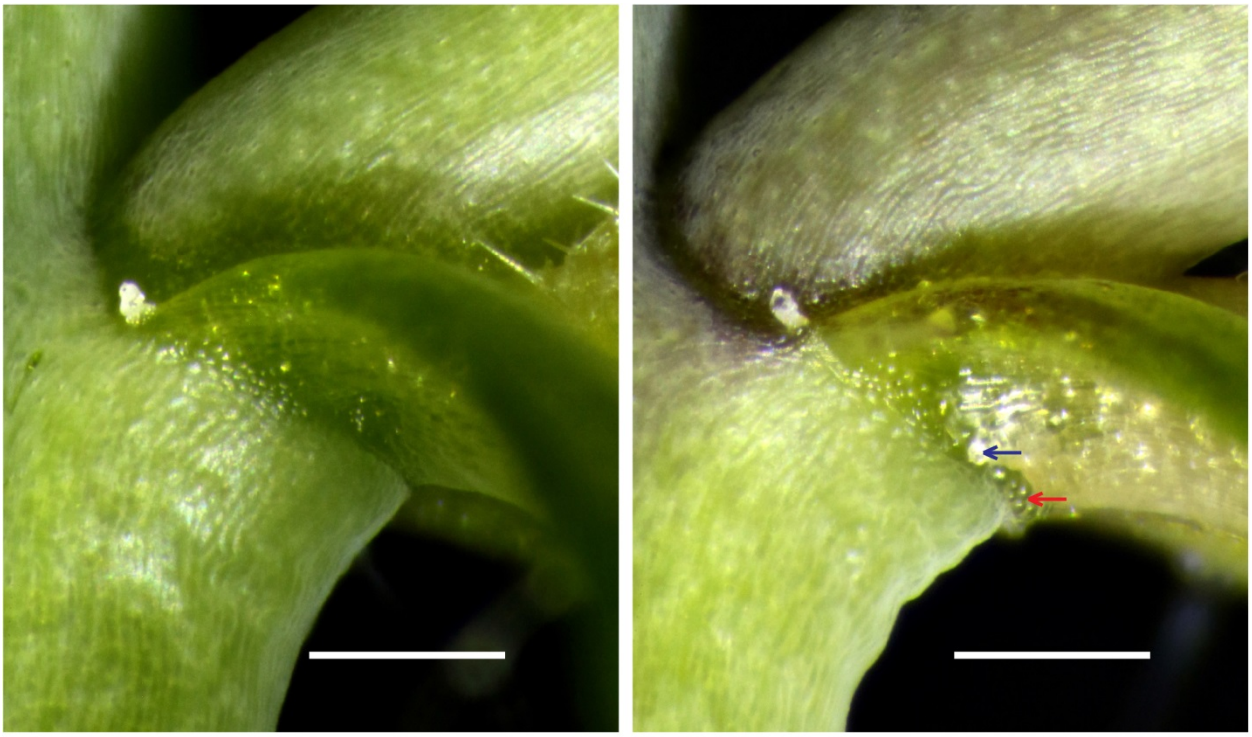

S4 Fig. **Enlargement of control MgCl$_2$ treatment and distal half DC3000 infection (half away) from figure 3**. Blue arrow indicates the area where cell separation has occurred. Red arrow indicates the swollen abscission zone cells.
